# Supplementary material for: Multi-state model for predicting ocular progression in acute Stevens-Johnson syndrome/toxic epidermal necrolysis
Source: PLoS One. 2021 Dec 23;16(12):e0260730. doi: 10.1371/journal.pone.0260730 (PMC8716030; doi:10.1371/journal.pone.0260730)
Supplement: S1 Table — (DOCX) [file pone.0260730.s001.docx]

**S1 Table.** Systemic Severity Index Score for SJS/TEN

| Evaluated Condition | Score |
| --- | --- |
| **1. Mucous lesions** |  |
| Ocular lesions |  |
| Pseudomembrane formation | 1 |
| Ocular surface epithelial defect (Ocular surface erosive lesions) | 1 |
| Bilateral acute keratoconjunctivitis | 1 |
| Labial and/or oral lesions |  |
| Oral diffuse erosive lesions with bloody scales | 1 |
| Labial erosive lesions with bloody scales alone | 1 |
| Oral or labial erosive lesions alone | 1 |
| Genital involvement | 1 |
| **2. Body surface area of skin lesions** (select 1 from the 3) |  |
| ≥30% | 3 |
| 10%–30% | 2 |
| <10% | 1 |
| **3. Fever:** ≥38.0°C | 1 |
| **4. Respiratory dysfunction** | 1 |
| **5. Epidermal detachment** | 1 |
| **6. Liver dysfunction** (Alanine aminotransferase ≥ 100 IU/L) | 1 |

Summed scores of ≥ 6 were treated as severe, while summed scores of ≤ 5 were treated as moderate. Each of the following conditions was evaluated as severe, regardless of the summed score: (1) pseudomembrane formation and/or ocular surface epithelial defect, (2) respiratory dysfunction associated with SJS/TEN, (3) TEN extending with diffuse erythema.

SJS: Stevens-Johnson syndrome; TEN: Toxic epidermal necrolysis
